# *Echinacea angustifolia* DC. Lipophilic Extract Patch for Skin Application: Preparation, In Vitro and In Vivo Studies

**DOI:** 10.3390/pharmaceutics12111096

**Published:** 2020-11-16

**Authors:** Dritan Hasa, Simon Žakelj, Iztok Grabnar, Francesco Cilurzo, Stefano Dall’Acqua, Antonella Riva, Beatrice Perissutti, Dario Voinovich

**Affiliations:** 1 Department of Chemical and Pharmaceutical Sciences, University of Trieste, P. le Europa 1, 34127 Trieste, Italy; dhasa@units.it; 2 Faculty of Pharmacy, University of Ljubljana, Aškerčeva 7, SI-1000 Ljubljana, Slovenia; simon.zakelj@ffa.uni-lj.si (S.Ž.); iztok.grabnar@ffa.uni-lj.si (I.G.); 3 Department of Pharmaceutical Sciences, University of Milano, Via G. Colombo, 71-20133 Milano, Italy; francesco.cilurzo@unimi.it; 4 Department of Pharmaceutical Sciences, University of Padova, Via F. Marzolo 5, 35131 Padova, Italy; stefano.dallacqua@unipd.it; 5 R&D Indena SpA, Viale Ortles, 12, 20139 Milano, Italy; antonella.riva@indena.com

**Keywords:** *Echinacea angustifolia* lipophilic extract, tetraene, drug-in-adhesive patch, in vitro skin permeation, pharmacokinetics

## Abstract

Dodeca-2*E*,4*E*,8*Z*,10*E*/*Z*-tetraenoic isobutylamide (tetraene) is the main component of *Echinacea angustifolia* DC. lipophilic extract, the bioavailability and immunomodulatory effect after oral administration in soft gel capsules in healthy volunteers of which we have already demonstrated. In the present work, we assessed the transdermal administration as an alternative route of administration of such an alkamide. The first step, therefore, encompassed the preparation of a drug-in-adhesive patch with an area of 868 mm^2^ and containing a dose of 0.64 mg of tetraene. In vitro skin permeation studies in Franz-type diffusion chambers resulted in a tetraene flux of (103 ± 10) ng × cm^−2^ × h^−1^ with a very good linearity (r = 0.99). The relatively low lag time of just 13 min indicates low binding and the accumulation of tetraene in the skin. Finally, the patch was administered to six healthy volunteers, and the pharmacokinetic analysis was performed by nonlinear mixed effects modelling with soft gel oral capsules serving as the reference formulation. The in vivo results correlated well with the in vitro permeation and indicated an initial burst tetraene absorption from the patch that was in parallel with the zero-order kinetics of absorption. The rate of the latter process was in good agreement with the one estimated in vitro. The tetraene absorption rate was therefore slow and prolonged with time, resulting in a bioavailability of 39% relative to the soft gel capsules and a very flat plasma concentration profile.

## 1. Introduction

Transdermal patches are self-contained discrete dosage forms that deliver drugs through the skin for systemic effects at a predetermined and controlled rate. They have several peculiar advantages over oral administration including the absence of first-pass liver metabolism, food interactions, gastrointestinal side effects, and easy, controlled dosing with constant plasma concentrations. The occlusive effect of the transdermal patches also facilitates the hydration of stratum corneum and consequently accelerates the delivery of the active ingredients to skin. The patch provides a series of additional benefits, such as the ease of use by simply adhering to the skin, portable packaging for active lifestyles, and limited irritation, thus resulting in improved patient compliance. Generally, there are two concepts of transdermal patches: the matrix and reservoir types, differing for the incorporation of the active agent. For the matrix type, the active ingredient is dispersed in a rate-controlling polymer, while in the reservoir type, a rate-controlling membrane contains the core where the active pharmaceutical ingredient is dispersed or solubilized.

In the pharmaceutical field, the use of transdermal patches is large, both for research purposes and for marketing new delivery systems [1,2]. Applications of transdermal patches for the delivery of herbal medicines or extracted herbs are less common, although some can be found. Actually, administration through the skin in the case of herbal drugs or plant active ingredients should be particularly noted because it is well known that such types of products suffer from instability in acidic pH, liver metabolism, interaction with food, and limited absorption due to their physicochemical characteristics, all of which often result in reduced oral bioavailability of such herbal medicines.

Transdermal patches have been developed for the delivery of different kinds of extracts: dry extracts of a single herbal drug, such as ginger [3], tamarind [4], soy extract [5], dry polyherbal extracts [6,7], lipophilic extracts such as ginger [8] or *Curcuma longa* [9], and fluid extracts of green tee [10] or *Momordica charantia* [11]. Examples of the marketed transdermal plasters containing herbal medicines include products delivering mixtures of natural ingredients for weight control or pain relief [12].

The aim of this study was therefore to investigate the feasibility of the transdermal delivery for the major alkamide of lipophilic *Echinacea* extract. The administration of dodeca-2E,4E,8Z,10E/Z-tetraenoic isobutylamide (tetraene) via the skin would have numerous particular advantages, including elimination of first-pass liver metabolism and food interactions, as well as easy, controlled dosing with constant plasma concentrations. Alkamides are the major lipophilic constituents of *Echinacea* preparations that are widely used in some European countries and in North America for common colds [13]. Previously published in vivo data in humans suggest that the major alkamide, tetraene, plays a very important role due to its numerous immunomodulatory and anti-inflammatory properties via the modulation of macrophages and PMN immune cells and cytokine/chemokine expression [14,15,16,17]. The cutaneous application of *Echinacea* lipophilic extract appears of interest because 2*E*,4*E*,8*Z*,10*E*/*Z*-tetraenoic acid isobutylamide possesses appropriate physicochemical properties for transdermal delivery. In fact, tetraene is a low molecular-weight molecule, i.e., less than 250 g/mol, with a limited solubility in water [18]. 

A drug-in-adhesive patch formulation containing the alkamide was prepared and evaluated by in vitro skin permeation through human epidermis to screen its ability to favor the partition of the active ingredient towards the stratum corneum and to provide an indirect measurement of its thermodynamic activity in the adhesive matrix as required by the EMA Guideline on Quality of transdermal patches (EMA/CHMP/QWP/608924/2014). The patch was then administered to six healthy volunteers by means of a one-day exposure. In all samples, tetraene concentrations were quantified by means of an LC–MS/MS analysis. Finally, a pharmacokinetic analysis was performed by nonlinear mixed effects modelling with soft gel oral capsules, as studied in a previous work [17] and serving as the reference formulation. 

## 2. Materials and Methods 

### 2.1. Materials

An *Echinacea angustifolia* DC lipophilic extract enriched with alkamides containing 10%. *w*/*w* of dodeca-2*E*,4*E*,8*Z*,10*E*/*Z*-tetraenoic isobutylamide, commonly known as tetraene (Indena, Milan, Italy); 25 μm-thick polyethylene/polyurethane coupled film (Rossella, Varese, Italy) as the patch supporting layer; Duro-Tak 380-3954 (Henkel Corporation, Bridgewater, NJ, USA) as the adhesive acrylic matrix; white paper coated on one side with silicone (Rossella, Varese, Italy) as the liner; acetonitrile (LC–MS grade, Rotisolv®, Carl Roth GmbH., Karlsruhe, Germany); Tris buffer–Pufferan (Carl Roth GmbH., Karlsruhe, Germany), ethyl acetate, formic acid, and methanol (BDH, Liverpool, UK); Tris buffer (Tris-d11, 99% D); sodium chloride, disodium hydrogen phosphate, potassium dihydrogen phosphate, and benzanilide (98%) (Aldrich, Milan, Italy); and (2*E*,4*E*,8*Z*,10*E*/*Z N*-isobutil-2,4,8,10-dodecatetraenammide (Phytolab GmbH, Vestenbergsgreuth, Germany) as the analytical standard. All the solvents, unless otherwise specified, were of analytical grade. A Barnstead Easy pure^®^ RF (Barnstead Thermolyne, Dubuque, IA, USA) compact ultrapure water purification system was used to obtain the purified water for the LC–MS/MS analysis.

Soft gel capsules, previously studied in [17] and serving as the reference formulation, contained 10 mg of the above-mentioned extract corresponding to a dose of 1 mg of dodeca-2*E*,4*E*,8*Z*,10*E*/*Z* -tetraenoic isobutyl amide (tetraene). The capsules, each weighing 24 mg, also contained the following inactive ingredients: gelatin, glycerin, titanium dioxide, and iron oxide yellow (Pharmagel Engineering S.p.A. Lodi, Milano, Italy). 

### 2.2. Methods

#### 2.2.1. Patch Preparation and Characterization 

Matrix patches were prepared by the casting and drying technique using a lab-scale coating unit Mathis LTE-S (M) (Oberhasli; Zurich, Switzerland). The composition of the adhesive matrix was 70.60% Duro-Tak 380-3954 and 29.40% *Echinacea angustifolia* lipophilic extract (*w*/*w*). Each 30 × 30 mm patch had an 868 mm^2^ surface area and was loaded with 6.5 mg of lipophilic extract.

Samples, with dimensions of 2.5 × 2.5 cm, of the patch (TW), the backing layer (TBL), and the release liner (TRL) were placed between the jaws of the MI 1000 micrometer (ChemInstruments, Fairfield, OH, USA), and the thickness of each was measured. The thickness of the matrix layer was calculated as the difference of TW − (TBL + TRL). The result is expressed as a mean of 5 measurements.

#### 2.2.2. Tetraene Patch Content 

A patch sample was dissolved in 10 mL of HPLC-grade ethanol. The solution was sonicated, filtered through a 0.45 μm filter, and assayed by an LC–MS/MS analysis using a previous method [17]. The results are expressed as mean ± SD (*n* = 3).

### 2.3. In Vitro Skin Permeation Study

The permeation studies were performed using abdominal skin from donors who underwent cosmetic surgery. According to an internal protocol [19], after removing the subcutaneous fatty tissue, the skin samples were immersed in water at 60 °C for 1 min, and the epidermis was carefully removed from the underlying tissue with the help of forceps. The integrity of epidermis samples was assessed by measuring their electrical resistance (voltage: 100 mV, frequency: 100 Hz; Agilent 4263B LCR Meter, Microlease, I) using a modified Franz diffusion cell (PermeGear, inc., Hellertown, PA, USA) with an effective permeation area and receptor volume of 0.636 cm^2^ and 3 mL, respectively [20]. Samples with an electrical resistance higher than 30 kΩ·cm^2^ were used. 

At the beginning of the experiment, a 2.5 cm^2^ circular sample was applied to the stratum corneum face of the epidermis specimen, and the assembly was mounted onto the receiver compartment of the Franz diffusion cell maintained at 35 ± 1 °C so that the skin surface temperature was 32 ± 1 °C. Special care was taken to avoid air bubbles between the epidermis and the medium in the receptor compartment. The receptor medium was continuously stirred with a small magnetic bar at 1800 rpm to assure a uniform distribution of the permeated drug.

The upper and lower parts of the Franz diffusion cell were sealed with Teflon (VWR International, I) and Parafilm^®^ (Pechiney Plastic Packaging Company, Chicago, IL, USA), and they were fastened together by means of a clamp. 

The receiving solutions were withdrawn at 5 time-points (1, 3, 5, 7, and 24 h) and quickly replaced with the same volume (0.2 mL) of fresh receiver medium. Sink conditions were maintained throughout the experiments. The withdrawn samples were directly assayed by LC–MS/MS. 

The cumulative amount of tetraene (Q) permeated through the human epidermis per unit of area was calculated from the concentration the receiving medium and plotted as a function of time. The steady flux (J) was calculated as the slope of the linear portion of the plot. 

### 2.4. In Vivo Studies

#### 2.4.1. Study Design

Six healthy volunteers of both genders (4 males and 2 females) between 33 and 58 years of age participated in this study. All the subjects had normal liver function and no diagnosed allergy or sensitivity to Compositae or Grossulariaceae plants. None of the volunteers were on a special diet. No medicines were taken during the study, and the volunteers were asked not to smoke or eat, as well as drink only water for 12 h before administration. All subjects gave their informed written consent to participate in this study. After an overnight fasting, a transdermal patch containing a dose of 0.64 mg of tetraene was applied to the forearm on each healthy volunteer. Blood samples (5 mL) collected in heparinized tubes were taken at 0 (before administration) and 1, 3, 5, 7, and 24 h after each dose. Plasma was immediately separated by centrifugation and stored frozen at −80 °C for analysis. The study protocol was approved by the University of Trieste Human Research Ethics Committee (permission no. 14/08). 

#### 2.4.2. Plasma Samples Preparation

The plasma samples were extracted using a solid-phase extraction technique [14,16]. Briefly, 2 mL of the Tris buffer and 100 µL of benzanilide solution, used as internal standard (I.S.), were added to 2 mL of plasma and vortexed for 1 min. Subsequently, samples were centrifuged for 10 min at 3200 rpm using Eppendorf 5810R centrifuge (Hamburg, Germany). The supernatant was applied onto 100 mg Isolute C18 columns from International Sorbent Technology (Mid Glamorgan, UK) and pre-treated with 1 reservoir volume (RV) acetonitrile followed by 1 RV water. The C18 cartridges were placed on a VacMaster sample processing station and subsequently washed with 1 RV of water under vacuum. The dodeca-2*E*,4*E*,8*Z*,10*E*/*Z*-tetraenoic acid isobutyl amides were eluted with 2 mL of acetonitrile. The eluents were evaporated under a stream of nitrogen at 40 °C using a TurboVab LV vaporator (Zymark, Hopkinton, MA, USA), the dry residue was dissolved in 100 µL of acetonitrile:water (6:4), and 20 μL were used for the LC/MS analysis.

#### 2.4.3. LC–MS/MS Analysis of Tetraene

Tetraene concentrations in both the patches and plasma samples were determined by the LC-MS analysis, as proposed by [14] and previously used in [16,17,21].

### 2.5. Pharmacokinetic Analysis

To get an insight into the rate and extent of tetraene transdermal absorption, pharmacokinetic data were analyzed using a population pharmacokinetic modelling approach. Mixed effects modelling and NONMEM software, version 7.4 (Icon Development Solutions, Ellicott City, MD, USA) was used for the analysis. Pharmacokinetic data from our previous study with the oral administration of *Echinacea angustifolia* lipophilic extract formulated as soft gel capsules were used as a reference [17]. This was a single-dose study in 10 healthy subjects, and the dose of Echinamide was 1 mg. The absolute bioavailability of Echinamide following oral administration is unknown; however, based on comparison of Echinamide exposure expressed as the area under the curve at various doses [17], we have estimated that it is maximum 20%. All data (oral and transdermal patch) were analyzed simultaneously. Various disposition models (one- and two-compartment models with first-order elimination) and various absorption models (first- and zero-order, with and without lag-time and combinations thereof) were tested. Between-subject variability was modeled with an exponential model. Additive, proportional, and combination (additive and proportional) error models were evaluated for the residual unexplained variability. A first-order conditional estimation method with interaction was used for parameter estimation. Model development was guided by the law of parsimony, giving the preference to the simplest model to adequately describe the data. Model selection was based on the objective function value (OFV), the precision of parameter estimates, and standard goodness-of-fit diagnostic plots. The convergence of minimization, the number of significant digits more than 3, successful covariance step, gradients in the final iteration between 10^−3^ and 10^2^, and parameter shrinkage were also considered. 

## 3. Results

Patches, uniform in color and aspect, without any evidence of phase separation over the period of the study, were successfully prepared with *Echinacea angustifolia* lipophilic extract containing 0.64 ± 0.01 mg of tetraene for each single dose (patch with an area of 868 mm^2^). The thickness of the adhesive matrix was 0.074 ± 0.000 mm (mean ± SD; *n* = 5).

### 3.1. In Vitro Transdermal Permeability

**Figure 1:**
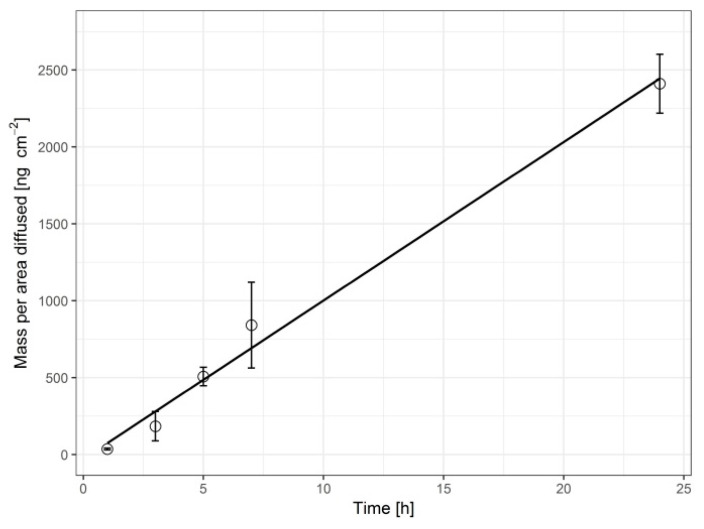
Tetraene mass vs time diffusion plot as a result of Franz diffusion chamber experiments (mean ± SD; *n* = 3).

The Franz diffusion chamber experiments resulted in a tetraene flux of (103 ± 10) ng × cm^−2^ × h^−1^ with a very good linearity (r = 0.99) of the mass permeated vs. time plot (Figure 1) and a mean lag time of only 13 ± 56 min. The in vitro experiment ended with 3.3% (at time = 24 h) of the available dose permeated through the skin barrier into the acceptor compartment. An apparent permeability coefficient P_app_ = (0.029 ± 0.003) nm/s could also be calculated because of the observed linearity and constant donor (patch) concentration. 

### 3.2. In Vivo Phamacokinetic Study

An exploration of the pharmacokinetic profiles revealed significant difference in the terminal slopes after dermal (0.069 ± 0.024) h^−1^ versus oral (0.541 ± 0.164) h^−1^ administration (mean ± SD, *p* < 10^−5^, Welch’s *t*-test), thus indicating prolonged transdermal absorption. One could also see an initial burst that suggested fast absorption immediately after administration. The maximum observed plasma concentration (C_max_) after patch administration was (0.748 ± 0.233) ng/mL at (0.33 ± 0.82) h (individual profiles are reported in Supplementary Materials Figure S1).

Absorption after oral administration was variable, with double peaks in the majority of the individual profiles (Figure S2). Moreover, in some subjects, the first peak was predominant over the second, while in others, it was the opposite. This double peak phenomenon was modelled as absorption from two compartments with the lag-time and variable ratio of the amount of the drug absorbed by the two pathways. 

The final disposition model was a one compartment model, and the estimated parameters were oral clearance (CL/F) and the volume of distribution after oral administration (V/F), where F is the absolute bioavailability after oral administration. Parameters related to the oral absorption were the absorption rate constants from compartments one and two (Ka_1_ and Ka_2_, respectively), absorption lag-time from compartment two (t_lag2_), and the fraction of the dose absorbed from compartment one (F_1_). It is noteworthy that the fraction of the bioavailable dose absorbed from compartment two was equal to 1-F_1_. Transdermal absorption was described by a two-part model consisting of the first- and zero-order process in parallel. The estimated parameter was the-first-order absorption rate constant (K_td1_). Additional parameters were the fraction of the bioavailable dose absorbed by the first order process (F_td1_) and the relative bioavailability of transdermal versus oral administration (F_r_). The parameters of the final model are summarized in Table 1.

Based on the estimated parameters related to the zero-order absorption from the patch, we calculated the total amount of tetraene absorbed by this process. Dividing this amount by 24 h (duration of exposure) and by the surface area of the patch (*S*), we calculated the flux (*j*) of 72 ng × cm^−2^ × h^−1^ according to Equation (1):

(1) $$j=\frac{\left(1-F_{td1}\right)F_{r}\;F\;Dose}{24\;S}.$$

**Figure 2:**
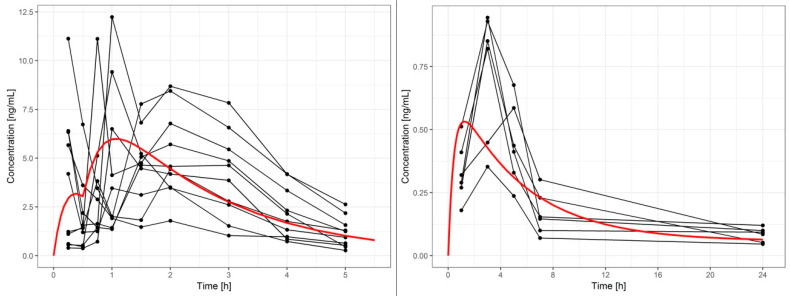
Plasma concentration profiles of tetraene after an oral dose of 1 mg (**left**) and a transdermal dose of 0.64 mg (**right**). Circles—measurements; black lines connect the same subject. Plasma concentration–time course in a typical subject (population prediction) is shown by the red curve.

The pharmacokinetic profiles of tetraene following oral and transdermal administration are shown in Figure 2, Figure S1 and S2.

**Figure 3:**
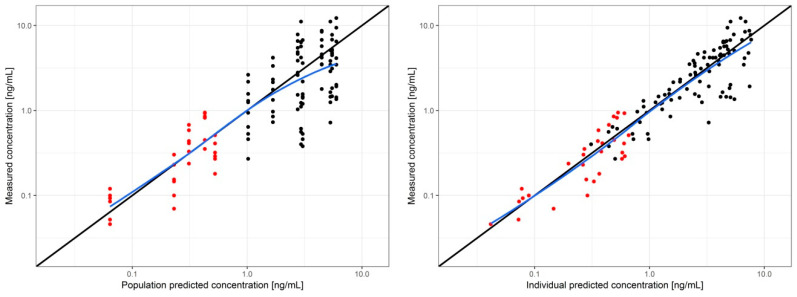
Goodness of fit plots of the final pharmacokinetic model of tetraene. Circles—measured versus population predicted concentration (**left**) and measured versus individual predicted concentration (**right**) after transdermal (red) and oral (black) administration. Black line—identity line; blue line—loess fit.

Goodness of fit plots (Figure 3) demonstrate no evident trend of model misspecification.

## 4. Discussion

The apparently low in vitro permeability is in line with other drug products available on the market such as diclofenac patches [22].

**Figure 4:**
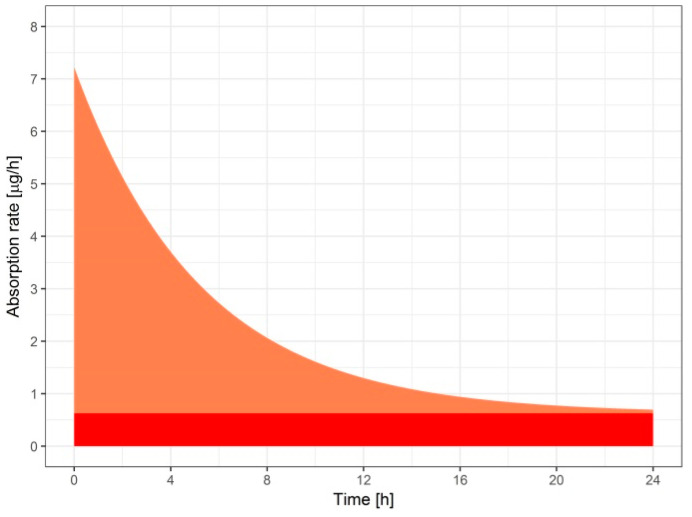
Absorption rate from the patch in vivo as a function of time estimated by the pharmacokinetic model. The pharmacokinetic modelling analysis indicated parallel zero-order (red) and first-order (coral) processes.

The expected in vivo consequence of the steady flux of tetraene was a prolonged exposure to the relatively low concentration of the active ingredient. Additionally, the low lag-time indicated a low binding and accumulation of the tested compound in the stratum corneum that could be expected due to the high lipophilicity of the compound [23]. Indeed, the mean in vivo human plasma concentrations of tetraene from the same formulation remained above 0.08 ng mL^−1^ with a C_max_ of (0.748 ± 0.233) ng mL^−1^ for the 24 h sampling period (Figure 2). Fitting the results of the pharmacokinetic models tested to the in vivo data for the transdermal absorption from the patches indicated an interesting combination of absorption processes. Specifically, a slow zero order absorption process continued the entire time of patch adhesion and delivered 30% of the absorbed tetraene in a steady flux of 72 ng × cm^−2^ × h^−1^, which very nicely correlated with the value established in vitro, as shown above (103 ng × cm^−2^ × h^−1^). Simultaneously, the majority (70%) of the absorbed tetraene appeared to be delivered from the patch via an absorption process governed by first order kinetics with a half-life of 3.5 h (Table 1). This provided the burst in tetraene absorption presented in Figure 4. The burst was absent in the in vitro data at all, and we therefore assume it was triggered in vivo, possibly by a mechanical stimulus such as the body movement of the volunteers who were not immobilized but rather followed their daily routines during the experiment to provide more realistic information.

While a direct mathematical connection between the in vitro diffusion and in vivo absorption assuming similar flux of a tested compound is often not realistic, the agreement between the in vivo zero-order and in vitro flux tempts us to estimate the missing pharmacokinetic parameters of tetraene based on previous work with the oral absorption of the same tested compound from soft gel capsules. The transdermal absorption pathway does not include significant pre-systemic metabolism or active elimination such as entero-enteric or entero-hepatic circulation, which could be indicated by the plasma concentration vs. time profile shown previously for oral administration pathway [17]. Therefore, the zero-order transdermal flux of the tested compound was similar to a constant infusion with the resulting plasma concentration obtainable by multiplying the measured flux with the area of the transdermal patch and dividing this product by the plasma clearance of the tested drug. The relative plasma clearance of tetraene (Cl/F) for oral administration was admittedly the only directly measured in vivo parameter available, but it was also established that the AUC/dose ratio that equaled F/Cl increased linearly with the dose given to an extent indicating that the F for the 1 mg soft gel capsules was likely at least five-fold lower compared to the highest reported dose given to humans [17], assuming a dose-independent clearance [24]. Therefore, the otherwise missing absolute F value was lower than 0.2, and the plasma concentration expected only from the zero-order diffusion from the transdermal formulation would be at least 0.06 ng mL^−1^, which is about half of the 24 h values measured by in vivo testing of the patch. As shown in Figure 3, the total plasma concentrations were much higher soon after the adhesion of the patches, primarily due to the initial absorption burst. Additionally evident from Figure 2 is that the peak plasma tetraene concentrations after transdermal applications were lower compared to those after oral application. Besides the more sustained release and slower absorption through the transdermal pathway, the difference was also due to the lower transdermal dose (0.64 vs. 1.0 mg) and lower bioavailability (39% of the soft gel bioavailability). The *Echinacea* extract’s concentration–immunomodulatory effect relationship is not yet fully understood, and it even appeared independent of the administered dose of alkamides in our previous study [16], as well as in the earlier work by Woelkart et al., where very similar concentrations to those achieved by the transdermal application were shown to modulate the pro-inflammatory cytokines [15]. 

The major limitation of our study was its modest sample size. Consequently, due to a relatively complex pharmacokinetic model between subject variability, the majority of parameters could not be estimated. Despite this limitation, our model adequately described the mean trend observed in the data. An additional limitation is the fact that the absolute bioavailability of tetraene was just an estimate, as it could not be determined experimentally. Our results are therefore exploratory in nature and should be interpreted as hypothesis-generating. Nevertheless, the estimated absorption rate in vivo was in good agreement with the flux determined in vitro, which validates our model.

## 5. Conclusions

The results reported herein demonstrate that the adhesive patch is a suitable dosage form for the delivery of tetraene because it permitted a precise dose administration and a sustained release of the active agent for (at least) 24 h. The in vitro sustained release of tetraene from the transdermal patch followed a zero-order rate model. Interestingly, the initial stages of in vivo transdermal absorption closely followed a first-order rate equation, meaning that it was dependent on its concentration (burst effect due to the initial amount of tetraene loaded into the patch), whereas it fit zero-order kinetics for the absorption of the last 30% of drug absorbed. To the best of our knowledge, this study is the first reported example of the delivery of the main alkamide of *Echinacea angustifolia* lipophilic extract through the skin. We also anticipate that this study opens new possibilities for the development of innovative delivery systems containing *Echinacea*.

## Figures and Tables

**Table 1:** Pharmacokinetic parameters of the final model.

Parameter	Estimate	^1^ RSE (%)
CL/F (L/h)	54.8	16
V/F (L)	24.3	63
Oral absorption		
F_1_ (%)	18.2	18
K_a1_ (h^−1^)	3.05	40
K_a2_ (h^−1^)	0.498	16
t_lag2_ (h)	0.508	75
Transdermal absorption		
F_r_ (%)	38.8	31
K_td1_ (h^−1^)	0.190	40
F_td1_ (%)	69.8	14
^2^ Between subject variability		
BSV_CL_ (%, %)	21.2, (4)	36
BSV_Ka1_ (%, %)	239, (36)	40
^3^ BSV_F1_ (%, %)	12.7, (59)	116
^2^ Residual variability		
Proportional error (%, %)	44.9, (7)	5

^1^ RSE: relative standard error; ^2^ BSV: between subject variability, variability reported as % coefficient of variability with shrinkage in (parenthesis). ^3^ Logit transformed, variability reported as SD. CL/F: oral clearance; volume of distribution after oral administration; F_1_: fraction of the dose absorbed from compartment one; K_a1_ and K_a2_: absorption rate constants from compartments one and two, respectively; t_lag2_: absorption lag-time from the compartment two; F_r_: relative bioavailability of transdermal versus oral administration; K_td1_: the-first-order absorption rate constant; and F_td1_: the fraction of the bioavailable dose absorbed by the first order process.

## Supplementary Materials

The following are available online at https://www.mdpi.com/1999-4923/12/11/1096/s1, Figure S1 Individual plasma concentration profiles of tetraene after administration of transdermal patch in 6 subjects with predictions of the pharmacokinetic model (circles—experimental measurements; red line—population prediction; blue line—individual prediction). Figure S2. Individual plasma concentration profiles of tetraene after administration of soft gel capsules in 10 subjects with predictions of the pharmacokinetic model (circles—experimental measurements; red line—population prediction; blue line—individual prediction).
