# Supplementary material for: Echinacea angustifolia DC. Lipophilic Extract Patch for Skin Application: Preparation, In Vitro and In Vivo Studies
Source: Pharmaceutics. 2020 Nov 16;12(11):1096. doi: 10.3390/pharmaceutics12111096 (PMC7697022; doi:10.3390/pharmaceutics12111096)
Supplement: Supplementary file 1 [file pharmaceutics-12-01096-s001.pdf]

pharmaceutics

Supplementary Materials: *Echinacea Angustifolia* DC. Lipophilic Extract Patch for Skin Application: Preparation, in Vitro and in Vivo Studies

Dritan Hasa, Simon Žakelj, Iztok Grabnar, Francesco Cilurzo, Stefano Dall'Acqua, Antonella Riva, Beatrice Perissutti and Dario Voinovich

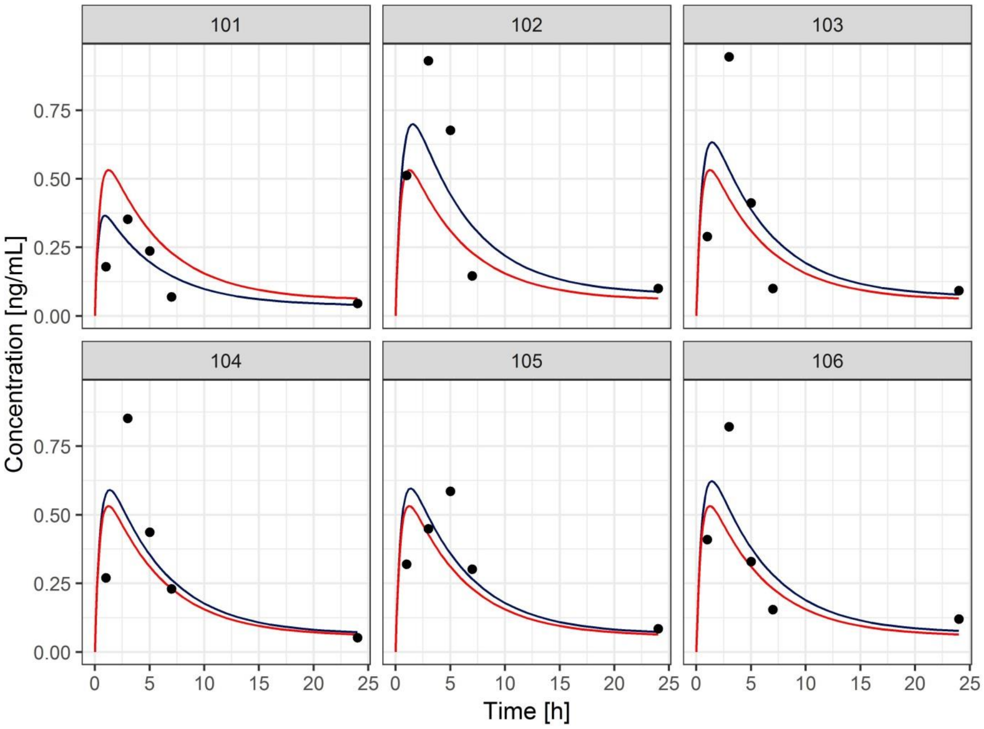

Figure S1. Individual plasma concentration profiles of tetraene after administration of transdermal patch in 6 subjects with predictions of the pharmacokinetic model (circles–experimental measurements, red line–population prediction, blue line–individual prediction).

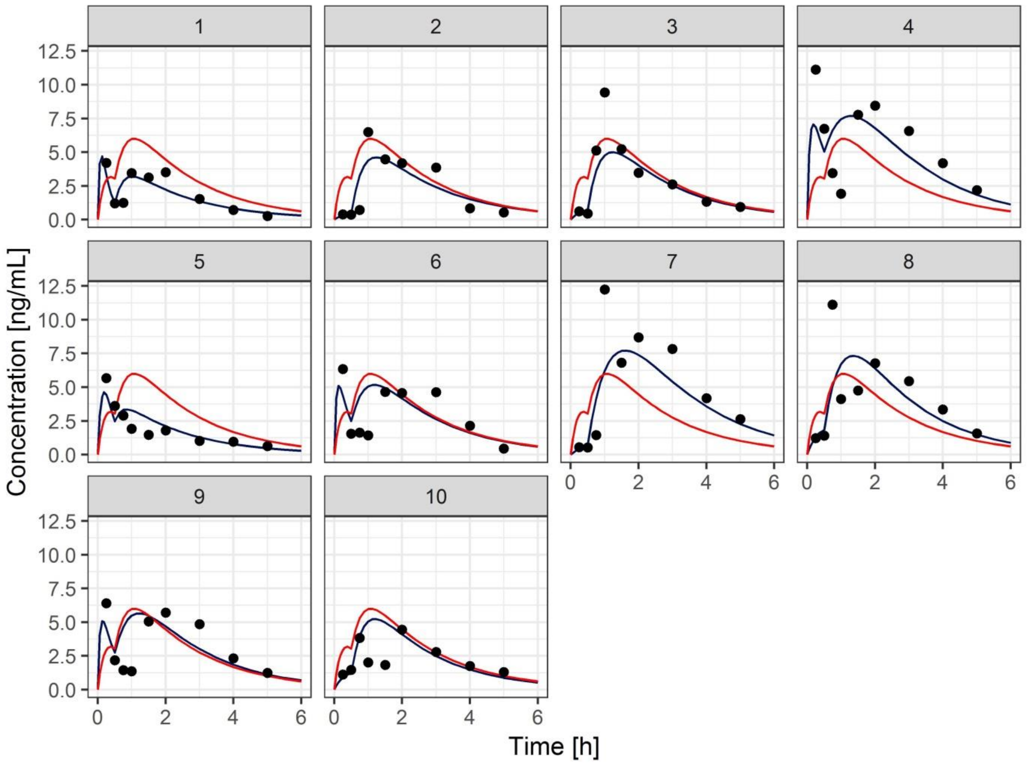

Figure S2. Individual plasma concentration profiles of tetraene after administration of softgel capsules in 10 subjects with predictions of the pharmacokinetic model (circles–experimental measurements, red line–population prediction, blue line–individual prediction).
